# ReCARving the future: bridging CAR T-cell therapy gaps with synthetic biology, engineering, and economic insights

**DOI:** 10.3389/fimmu.2024.1432799

**Published:** 2024-09-05

**Authors:** Alaa Ali, John F. DiPersio

**Affiliations:** ^1^ Stem Cell Transplant and Cellular Immunotherapy Program, Georgetown Lombardi Comprehensive Cancer Center, Washington, DC, United States; ^2^ Center for Gene and Cellular Immunotherapy, Washington University in Saint Louis, Saint Louis, MO, United States

**Keywords:** CAR T-cell therapy, immunotherapy, synthetic biology, gene editing, allogeneic CAR T cells, immunosuppressive microenvironment

## Abstract

Chimeric antigen receptor (CAR) T-cell therapy has revolutionized the treatment of hematologic malignancies, offering remarkable remission rates in otherwise refractory conditions. However, its expansion into broader oncological applications faces significant hurdles, including limited efficacy in solid tumors, safety concerns related to toxicity, and logistical challenges in manufacturing and scalability. This review critically examines the latest advancements aimed at overcoming these obstacles, highlighting innovations in CAR T-cell engineering, novel antigen targeting strategies, and improvements in delivery and persistence within the tumor microenvironment. We also discuss the development of allogeneic CAR T cells as off-the-shelf therapies, strategies to mitigate adverse effects, and the integration of CAR T cells with other therapeutic modalities. This comprehensive analysis underscores the synergistic potential of these strategies to enhance the safety, efficacy, and accessibility of CAR T-cell therapies, providing a forward-looking perspective on their evolutionary trajectory in cancer treatment.

## 1 Introduction

The concept of chimeric antigen receptor (CAR) T-cell therapy represents a transformative advance in cancer immunotherapy, bridging the innate power of cellular immunity with the precision of molecular targeting. The genesis of this revolutionary therapy dates back to the late eighties when the first CAR was engineered (1, 2), signifying the inception of a new era in targeted cancer therapy. These early constructs laid the foundational framework for what would become a series of iterative and transformative advancements in the field (3).

Over the ensuing decades, CAR T-cell therapy has evolved dramatically, propelled by significant technological innovations and a deeper understanding of cancer immunology. Initial clinical successes were most notable in hematologic malignancies, such as acute lymphoblastic leukemia (4, 5) and diffuse large B-cell lymphoma (6), where remission rates previously unattainable with traditional therapies were achieved. This was evidenced by landmark clinical trials that demonstrated profound responses in patients resistant to conventional treatments, establishing CAR T-cells as a pillar of modern oncological therapy (7–12).

Despite these impressive outcomes, expanding CAR T-cell therapy to broader oncological applications, particularly solid tumors, has encountered significant challenges (13). Key obstacles include the immunosuppressive tumor microenvironment (TME), antigen escape variants, and the physical barriers that impede CAR T-cell infiltration and function (13). Moreover, systemic toxicities such as cytokine release syndrome (CRS) and neurotoxicity pose severe risks, limiting the therapy’s widespread application (14, 15). Furthermore, the individualized manufacturing process for CAR T-cell therapy introduces logistical and economic hurdles (16), including high costs and variability in the quality of patients’ T cells.

**Figure 1:**
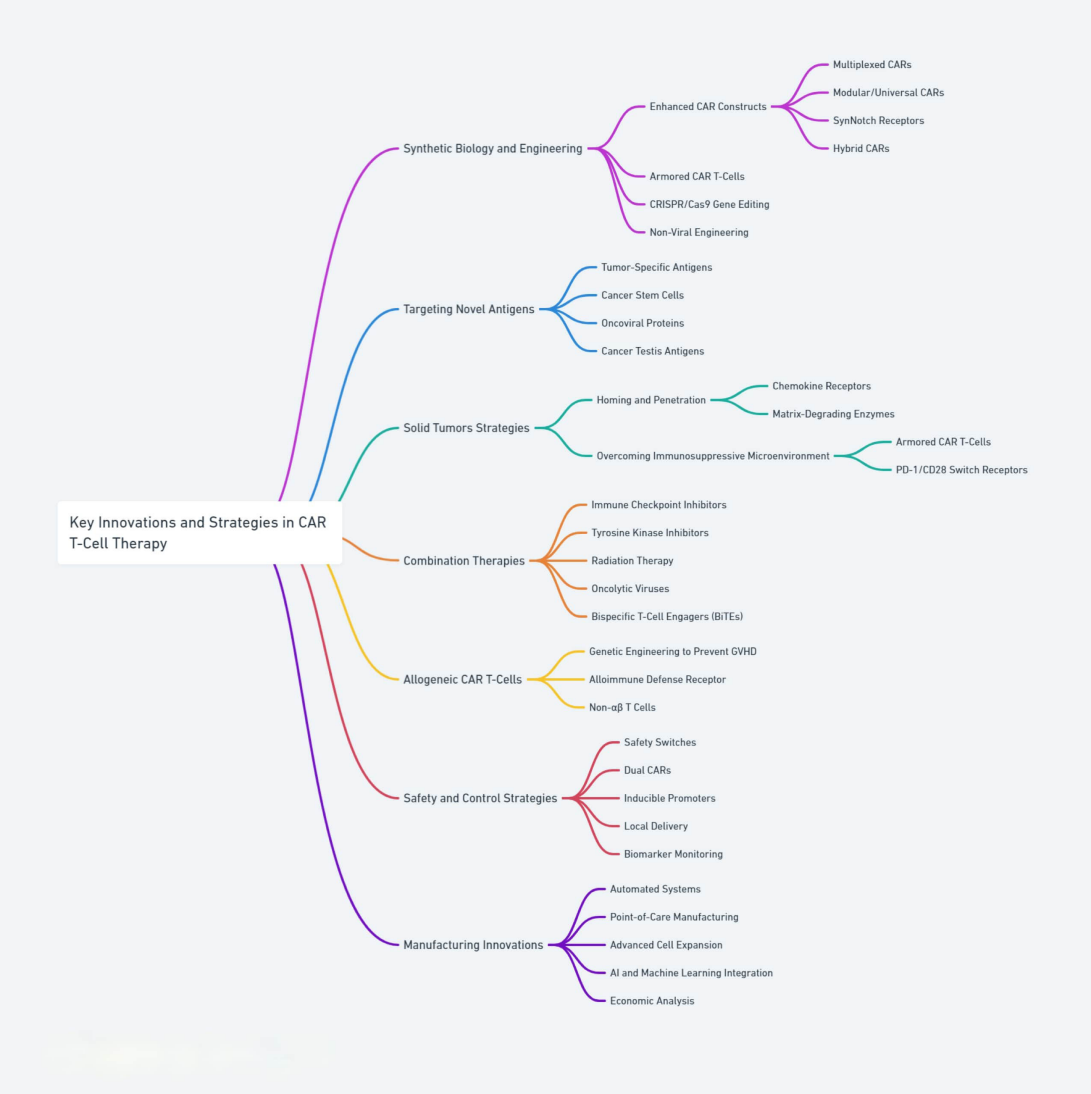
Core strategies and innovations in CAR T-cell therapies, highlighting advancements in design, engineering, and safety features.

Recognizing these hurdles, the scientific community has embarked on a quest to refine and evolve CAR T-cell strategies to overcome these barriers. This review examines emerging strategies shaping the future of CAR T-cell therapy, including next-generation CAR constructs, improved manufacturing processes, and novel combination therapies. It highlights the potential of these strategies to broaden the applicability of CAR T-cell therapies across a wider range of cancers providing a forward-looking perspective on their evolution in cancer treatment. Additionally, this review critically analyzes the field’s current gaps, controversies, and future directions, emphasizing the multifaceted challenges and opportunities in advancing CAR T-cell therapies **Table 1** and **Figure 1**.

**Table 1:** Innovative strategies in CAR T Cell Therapy.

	Strategy	Objective	Mechanism	Potential Outcomes
**Synthetic Biology and Engineering**	Multiplexed CARs 19, 20	Prevent tumor escape due to antigen loss or heterogeneity	Target multiple tumor-associated antigens to address antigen variability and loss	Enhanced tumor targeting and reduction in tumor escape mechanisms
	Modular/Universal CARs 21–26	Increase flexibility in targeting	Use of switchable, bispecific adaptors to redirect CAR T cells against various tumor antigens	Greater adaptability and precision in targeting tumors
	Synthetic Notch (SynNotch) CARs 27–29	Enhance specificity and safety of activation	Use dual antigen recognition strategy to initiate CAR transcription only after interacting with a primary antigen	More precise tumor targeting, reduced off-tumor activity, enhanced safety, and reduced potential toxicities
	Hybrid CARs 30–36	Enhance targeting of cancer-specific antigens and reduce tonic signaling	Combine TCR and CAR features to recognize intracellular antigens presented by MHC molecules at ultra-low densities	Broadened therapeutic applicability, ability to target the entire proteome of cancer cells
	Armored CAR T cells 39–41	Enhance efficacy and persistence	CAR T cells engineered to secrete cytokines or express pro-inflammatory ligands	Increased persistence and efficacy, and modulation of the TME
**Manufacturing Advancements**	CRISPR/Cas9 gene editing 42–44	Enhance CAR T cell functionality and persistence	Genetic modifications to secrete cytokines or express pro-inflammatory ligands	Improved anti-tumor activity, survival, and function in hostile tumor microenvironments
	Advancements in Vector Technology 46–47, 49–52	Improve gene delivery efficiency and safety	Optimization of viral vectors (lentiviral, retroviral) for stable gene transfer and reduced oncogenesis risk, non-viral methods (transposon-based systems, mRNA electroporation, CRISPR) for scalable and precise gene editing	Enhanced transduction rates, long-term CAR gene expression, increased safety and therapeutic efficacy
	Targeting specific T cell subsets 54, 55	Maximize therapeutic persistence and efficacy	Selection of Tcm and Tscm for longevity and potent antitumor responses, inclusion of both CD4+ and CD8+ T cells for synergistic effects	Enhanced long-term antitumor activity, durability, and therapeutic outcomes
	Optimizing Production and Reducing Costs 154–172	Streamline production, enhance efficiency, and reduce costs	Automated manufacturing systems, point-of-care production units, advanced cell expansion techniques, AI and ML integration, non-viral gene transfer methods, economic analysis, local manufacturing facilities, streamlined regulatory approvals	Reduced production time and costs, increased efficiency, improved accessibility, standardized treatments, expanded access in underserved regions, enhanced regulatory compliance
**Targeting Novel Antigens**	Tumor-specific and neoantigens 65–67	Expand efficacy across diverse cancer types	Identifying and targeting tumor-specific antigens (TSAs) and neoantigens unique to cancer cells	Minimized off-target effects, personalized treatment, broader therapeutic applicability
	Targeting cancer stem cells (CSCs) 75–77	Eradicate sources of tumor regrowth and metastasis	Engineering CAR T cells to recognize and eliminate CSC-specific antigens	More durable responses, reduced likelihood of cancer relapse
**Enhancing Solid Tumor Targeting**	Enhancing homing and penetration 78–82	Improve CAR T-cell trafficking and infiltration in tumors	Engineering CAR T cells to express chemokine receptors, incorporate matrix-degrading enzymes, and target tumor vasculature	Enhanced trafficking and infiltration, direct tumor starvation, boosted antitumor efficacy
	Overcoming the immunosuppressive TME 39–41, 83–88	Counteract the suppressive effects of the tumor environment	Development of "armored" CAR T cells, PD-1–CD28 switch receptors, knockdown of intracellular inhibitors, metabolic adaptations, and inhibition of tumor-derived exosomes	Improved CAR T-cell survival and function in hostile TME, enhanced anti-tumor activity
**Synergistic Combination Therapies**	Integration with Other Cancer Treatments 95, 96, 98–103, 108, 109, 111–115	Overcome barriers in immunotherapy and enhance CAR T cell efficacy	Combination with ICIs, TKIs, DNA damage repair inhibitors, angiogenesis inhibitors, low-dose chemotherapy, radiation therapy, oncolytic viruses, and BiTEs	Synergistic anti-tumor effects, enhanced CAR T cell functionality, better local control in solid tumors, reduced antigen escape
**Allogeneic CAR T-cell Therapy**	Allogeneic CAR T-cell Therapy 116–119, 121–125	Provide a standardized, ready-to-use treatment option	Use healthy donor T cells, bulk manufacturing, genetic modifications to prevent GVHD (e.g., TCR knockout, ADR integration)	Immediate availability, reduced manufacturing time and costs, consistent therapeutic outcomes
**Advanced Strategies to Mitigate Toxicities**	Enhancing Safety and Control 127–143	Reduce toxicities and enhance control in CAR T-cell therapies	Integration of safety switches (inducible caspase-9, ADCC switches), small molecule-based switches, SUPRA CARs, Dual CARs, inducible promoters, drug-responsive elements, sound/light activation	Rapid elimination of CAR T cells in severe side effects, precise targeting, reduced off-target effects, minimized risk of overactivation and associated toxicities
	Local delivery of CAR T cells 89–144	Minimize systemic exposure and reduce widespread toxicities	Administering CAR T cells directly to the tumor site	Reduced risk of widespread toxicities, enhanced local control of tumors
	Prophylactic Medications and Predictive Techniques^145^–^153^	Reduce severity of CRS and neurotoxicity, predict and manage toxic responses	Use of medications like tocilizumab and anakinra, biomarker monitoring, fractionated dosing, CRISPR/Cas9 gene editing, development of mouse models	Preemptive reduction of CRS and neurotoxicity, better prediction and management of toxic responses, identification of key inflammatory pathways, improved safety interventions

## 2 Core strategies and innovations in CAR T-cell therapy

### 2.1 Synthetic biology and engineering: redefining CAR T-cell design and manufacturing

Despite its promise, the efficacy of CAR T-cell therapy remains limited, with durable remissions being achieved in only 40% of diffuse large B-cell lymphoma patients (17). Enhancing the design of chimeric antigen receptor (CAR) constructs is a critical area of research aimed at augmenting the efficacy of CAR T-cell therapies. This effort involves the engineering of CARs with advanced signaling capabilities and the integration of safety features to address the limitations observed with first-and second-generation CAR T cells (18).

A range of innovative strategies focusing on the refinement of CAR constructs and optimization of manufacturing protocols have emerged. For example, multiplexed CARs targeting multiple tumor-associated antigens aim to forestall tumor escape mechanisms by addressing antigen loss or heterogeneity (19, 20). Similarly, the development of “modular” or “universal” CAR systems offers adaptable targeting capabilities through switchable, bispecific adaptors, enhancing the precision of CAR T-cell engagement with diverse tumor antigens (21–26). Furthermore, synthetic Notch (SynNotch) receptors represent a cutting-edge advancement in CAR T cell engineering, employing a dual antigen recognition strategy for activation. This novel approach uses SynNotch receptors to initiate transcription of a CAR after interacting with a predefined primary antigen, ensuring activation is strictly tumor specific (27–29). This two-step activation process allows for more precise tumor targeting and minimizes off-tumor activity, enhancing safety and reducing potential toxicities associated with conventional CAR T cell therapies. In addition to these innovations, Hybrid CARs technologies combine features from both T-cell receptors (TCRs) and CARs to enhance the targeting of cancer-specific antigens and reduce tonic signaling, enabling the engineered cells to recognize and engage with intracellular antigens presented at ultra-low densities on cancer cells by MHC molecules (30–36). This advancement will potentially broaden the therapeutic applicability of CAR technology, allowing it to target the entire proteome of a cancer cell.

Advancements in CAR T-cell design have led to the development of “third generation” CAR T cells, which represent a next-generation approach aiming to enhance the efficacy and longevity of these therapies. By integrating multiple co-stimulatory molecules into the CAR structure, these sophisticated constructs are designed to provide enhanced activation and sustained support to the CAR T cells (37, 38). Additionally, “armored” CAR T cells are genetically modified to enhance their efficacy and persistence. These modifications allow armored CAR T-cells to secrete active cytokines or express other pro-inflammatory ligands to enhance their anti-tumor activity, helping them better survive, disrupt, and modulate the tumor microenvironment, improving their function in hostile conditions (39–41).

In the pursuit of enhancing the efficacy and persistence of CAR T-cell therapies, researchers have innovated and refined manufacturing protocols. The integration of CRISPR/Cas9 gene editing represents a revolutionary stride in CAR T cell manufacturing. This precise gene-editing technology allows for the specific deletion of CAR T genes in an effort to bolster CAR T cell persistence and functionality, such as the knockout of genes encoding inhibitory receptors (42), epigenetic modifiers (43), and those mediating CAR T exhaustion (44). In parallel, advancements in vector technology have substantially improved the delivery of genetic material into T cells (45). Viral vectors, known for their high efficiency in gene delivery, have been optimized to enhance transduction rates while minimizing the risk of insertional oncogenesis—a concern with earlier-generation vectors especially retroviral vectors (46). Modern lentiviral and retroviral vectors offer stable gene transfer, which is crucial for the long-term expression of CAR genes (47). Non-viral engineering strategies have also progressed, offering more scalable and potentially safer alternatives to viral methods (48). These include transposon-based systems like Sleeping Beauty (49), which enable stable gene integration, and mRNA electroporation (50), which provides a transient expression that can be advantageous for safety. Additionally, non-viral methods such as the CRISPR complex delivery system for manufacturing CAR T cells offers enhanced safety by avoiding viral vector risks and ensuring precise gene editing without affecting other genome areas, thus minimizing off-target effects. The technology is also versatile, allowing for both gene knock-ins and knock-outs, enhancing the functional capabilities of CAR T cells and their therapeutic efficacy and safety (51, 52) **Table 2**.

**Table 2:** Comparison of viral, non-viral gene delivery, and CRISPR/Cas9 Gene Editing in CAR T-Cell Therapy.

Feature	Viral Gene Delivery	Non-Viral Gene Delivery	CRISPR/Cas9 Gene Editing
**Efficiency**	High transduction efficiency; stable gene expression.	Lower efficiency; often transient expression.	High editing efficiency; permanent modifications possible.
**Cost**	Higher due to production complexities and biosafety requirements.	Generally lower, simpler production processes.	Variable; high initial development cost but decreasing as technology matures.
**Safety**	Risk of insertional mutagenesis and immune response to viral components.	Reduced risk of insertional mutagenesis; lower immunogenicity.	Risk of off-target effects and unintended genetic alterations.
**Scale-Up**	Scalable but complex due to stringent regulatory requirements.	Easier to scale up and less regulated.	Scalable, but requires precise control and validation of editing tools.
**Flexibility and Control**	Less control over gene expression post-delivery.	Higher control, including potential for repeat dosing.	High precision in gene modification; allows targeted gene disruptions and insertions.
**Technological Maturity**	Well-established in clinical settings with approved products.	Emerging technologies, fewer examples of clinical validation.	Rapidly evolving; increasing clinical applications but still less mature than viral methods.
**Integration Into Host Genome**	Permanent integration possible, leading to long-lasting effects.	Usually no integration, leading to transient effects unless integrating non-viral systems are used.	Targeted integration can be achieved; depends on the CRISPR system and delivery method used.

The strategic selection of specific T cell subsets is pivotal in honing the efficacy of CAR T cell therapies. Central memory T cells (Tcm) and stem cell-like memory T cells (Tscm) are being employed due to their inherent longevity, robust proliferative abilities, and potent antitumor responses (53). These cells are known for their self-renewal capacity and long-term memory, providing a persistent immunological presence against tumors. Leveraging these subsets is instrumental in creating a pool of CAR T cells with superior proliferative capacity, long-term persistence, and potent antitumor activity (54, 55). Additionally, the inclusion of both CD4+ and CD8+ T cells could further enhance the therapeutic potential of CAR T-cell constructs. CD4+ T cells, often termed helper T cells, are crucial for their supportive role in immune modulation and enhancing the function of CD8+ T cells, which are primarily responsible for executing cytotoxic actions against tumor cells. Incorporating both subtypes not only facilitates a robust and sustained antitumor immune response but also capitalizes on the synergistic interactions between them to maximize therapeutic outcomes (56–58) **Table 3**.

**Table 3:** Comparison of CD4+ vs. CD8+ T Cell Subtypes in CAR T-Cell Therapy.

Feature	CD4+ T Cells	CD8+ T Cells
**Role in Immunity**	Primarily help activate other immune cells; provide support and enhance the immune response.	Primarily responsible for directly killing infected or cancerous cells.
**Outcomes in Therapy**	Enhance overall immune response, can contribute to more sustained disease control when included.	Often more effective at rapid tumor clearance; essential for immediate cytotoxic activity.
**Proliferative Capacity**	Generally lower proliferative capacity compared to CD8+ T cells but crucial for long-term immunological support and memory.	Higher proliferative capacity, crucial for immediate antitumor activity.
**Persistence**	Longer persistence in the body, which helps in maintaining a prolonged immune response against cancer cells.	Shorter persistence than CD4+ cells, but efforts to engineer longer-lasting CD8+ cells are ongoing.
**Therapeutic Efficacy**	Important for cytokine production and helping CD8+ T cells function optimally. Often engineered in CAR T-cell therapies for balanced responses.	Typically show higher efficacy in terms of direct tumor cell destruction in the short term. Used predominantly in most CAR T-cell constructs.
**Synergistic Potential**	Synergize with CD8+ T cells to enhance and sustain antitumor response. Can be engineered to help modulate the tumor microenvironment.	Synergy with CD4+ T cells enhances their effectiveness and longevity in the host.
**Clinical Implications**	Enhancements in CD4+ CAR T-cell designs are aimed at improving their antitumor functions and persistence, reflecting their role in achieving durable remissions.	Focus on enhancing the cytotoxic capacity and persistence to improve immediate and long-term clinical outcomes.

**Figure 2:**
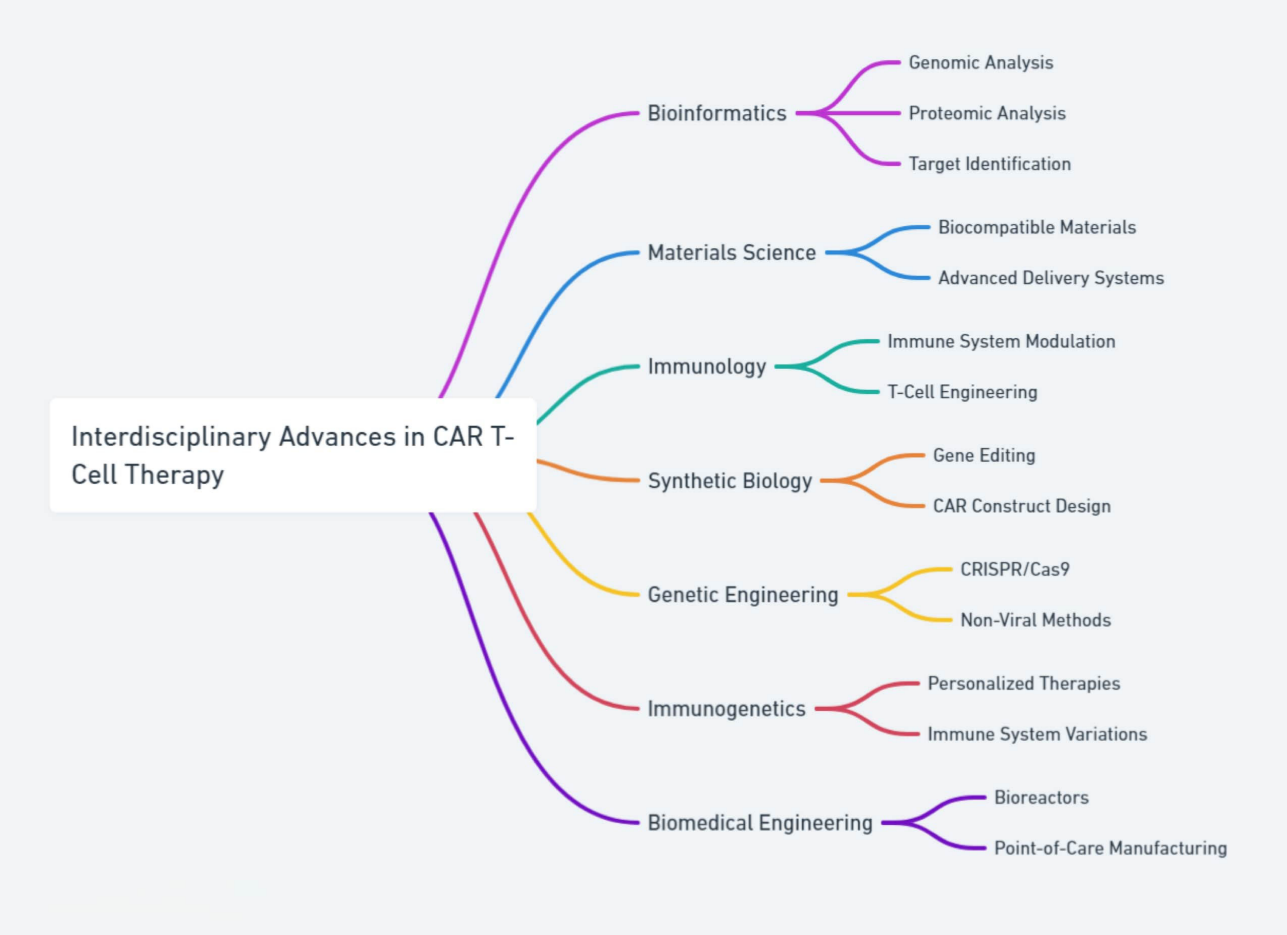
The integration of interdisciplinary approaches in advancing CAR T-cell therapy, combining insights from bioinformatics, materials science, immunology, single-cell studies, and omics technologies to enhance the precision and effectiveness of treatments.

Interdisciplinary approaches are crucial in advancing CAR T-cell therapy by integrating insights from bioinformatics, materials science, immunology, single-cell studies, and various omics technologies. Bioinformatics plays a pivotal role in analyzing large-scale genomic and proteomic data to identify new targets (59) and understand complex cellular behaviors (60, 61). Furthermore, single-cell technologies and omics analyses enable the detailed study of individual cell behaviors and responses, providing a more nuanced understanding of the variability and efficacy of CAR T-cell therapies (62). These interdisciplinary efforts are vital for tailoring therapies to individual patients’ unique biological contexts, ultimately enhancing the precision and effectiveness of CAR T-cell treatments (**Figure 2**).

The integration of synthetic biology and advanced engineering in CAR T-cell therapy brings forth transformative prospects for improving therapeutic efficacy and safety. However, this progress is accompanied by substantial challenges and controversies. Innovations such as multiplexed, modular, and SynNotch CAR systems provide unprecedented precision in tumor targeting, potentially reducing off-target effects and enhancing treatment specificity. Despite these advancements, the complexity and novel nature of these designs prompt concerns about their predictability and consistent performance in diverse clinical scenarios. The scientific community continues to debate the ideal balance between the sophistication of these designs and the practicalities of manufacturing, scalability, and regulatory approvals. Ethical considerations and the dynamic nature of regulatory standards further complicate the rapid adoption and implementation of these advanced therapies. Importantly, there is a critical need for comprehensive clinical trials to rigorously evaluate the long-term safety and efficacy of these next-generation CAR T cells. Such trials are essential to ensure that these innovative treatments can be safely integrated into clinical practice and can deliver sustained benefits to patients. As research progresses, streamlining manufacturing processes and establishing robust and more cost-effective regulatory frameworks will be pivotal in overcoming current limitations. Together, these steps will help to unlock the full potential of CAR T-cell therapies, extending their benefits beyond hematologic malignancies to include the effective treatment of solid tumors.

### 2.2 Expanding the horizon: targeting novel antigens for enhanced specificity and efficacy in CAR T-cell therapy

In the advancement of CAR T-cell therapy, a key focus has been on identifying and targeting new antigens to expand the therapy’s efficacy across diverse cancer types. Researchers are exploring tumor-specific antigens (TSAs) and neoantigens that are unique to cancer cells, minimizing off-target effects and personalizing treatment (63–67). Oncoviral proteins (68, 69) and cancer testis antigens (CTAs) and other embryonic antigens (70, 71) provide new targets, especially in cancers associated with viral infections and limited normal tissue expression, respectively.

Targeting cancer stem cells (CSCs) with CAR T cell therapy is an emerging strategy aimed at eradicating the root of tumor regrowth and metastasis. CSCs are elusive targets due to their low abundance and expression of conventional antigens, but their role in driving tumor progression and recurrence makes them logical targets (72, 73). CAR T cells are being engineered to recognize CSC-specific antigens to selectively target and eliminate these progenitor cells, potentially leading to more durable responses and reducing the likelihood of cancer relapse (74–77).

The pursuit of targeting novel antigens in CAR T-cell therapy represents a cutting-edge approach that holds promise for enhancing treatment specificity and expanding efficacy across diverse cancer types. While the use of tumor-specific antigens and neoantigens offers a pathway toward more personalized and less toxic treatments, significant challenges remain. Identifying and validating effective targets that are unique to cancer cells without affecting normal tissue is complex and requires rigorous testing. Furthermore, the targeting of cancer stem cells, though promising for preventing recurrence, presents issues of selectivity and safety due to their low abundance and heterogeneity. Continued advancements in antigen discovery, coupled with an integrative approach that combines CAR T-cell therapy with other treatment modalities, are critical for overcoming current limitations and fully realizing the potential of this powerful therapeutic tool.

### 2.3 Strategies to enhance CAR T-cell therapy in solid tumors: homing, penetration, and overcoming immunosuppressive microenvironment

Addressing the formidable challenges posed by solid tumors necessitates innovative strategies in CAR T-cell therapy, focusing on two critical areas: enhancing homing and penetration, and overcoming the immunosuppressive tumor microenvironment (TME). Solid tumors present a unique set of barriers, including a dense extracellular matrix and a hostile TME characterized by immune-suppressive cells and factors. To improve CAR T-cell homing, research has pivoted towards engineering CAR T cells to express specific chemokine receptors that match the chemokines secreted by tumors, thereby enhancing their trafficking and infiltration capabilities (78, 79). Furthermore, modifications are being explored to facilitate penetration through the tumor’s dense matrix by incorporating matrix-degrading enzymes into CAR T-cell designs (80, 81). Another emerging strategy is targeting tumor vasculature with CAR T cells engineered to attack specific endothelial markers which offers a dual benefit: direct tumor starvation and enhanced immune cell infiltration, boosting antitumor efficacy (82).

Simultaneously, strategies to counteract the immunosuppressive effects of the TME are critical. This includes the development of “armored” CAR T cells capable of secreting cytokines to modulate the TME, making it more conducive to T-cell activity, as discussed in the previous section (39–41). Additionally, various genetic modifications are being considered, including the design of PD-1–CD28 switch receptors (83), which are engineered to convert inhibitory signals into stimulatory ones, and the knockdown of intracellular inhibitors (84) to maintain CAR T-cell activation and effector functions. Metabolic adaptation is also key, where CAR T cells are engineered to withstand the nutrient deprived and hypoxic TME (85). Enhancements to the CAR T metabolic pathways enable them to maintain functionality and survive in the harsh tumor conditions (86, 87). In addition, there’s a growing interest in inhibiting tumor-derived exosomes, which often carry immunosuppressive molecules (88). By preventing these exosomes from reaching and impairing CAR T cells, it’s possible to preserve the T cells’ vigor and anti-tumor activity, further bolstering their effectiveness against solid tumors.

Local delivery of CAR T cells is an innovative method where CAR T cells are administered directly into the tumor site, offering a concentrated attack while potentially reducing systemic toxicity (89, 90). This targeted approach may improve the efficiency of CAR T-cell penetration and functionality within the solid tumor microenvironment. Advancing this concept, researchers are leveraging nanotechnology to develop novel delivery systems. These nano-carriers can protect CAR T cells during transit, enhance their migration and infiltration into tumors, and provide controlled release mechanisms, which could lead to improved persistence and efficacy of the CAR T cells (91, 92).

The innovative strategies being developed to enhance CAR T-cell therapy in solid tumors—focusing on improved homing, penetration, and overcoming the immunosuppressive microenvironment—represent significant advances in the field. While engineering CAR T cells to express specific chemokine receptors and matrix-degrading enzymes shows promise in enhancing infiltration into solid tumors, the complexity and heterogeneity of these tumors pose substantial challenges. The development of ‘armored’ CAR T cells and other genetically modified cells intended to modulate the hostile TME is progressing, yet concerns about safety, specificity, and long-term effects remain. Debates within the scientific community regarding the practicality of these complex interventions versus simpler, more robust approaches underscore the need for a balanced exploration of these technologies. Extensive clinical trials and continuous technological improvements are crucial to validate these strategies and ensure they can be safely and effectively integrated into patient care.

### 2.4 Synergistic combination therapies: enhancing CAR T-cell efficacy through multimodal treatment approaches

The integration of CAR T-cell therapy with other cancer treatment modalities is at the forefront of innovative strategies aimed at overcoming existing barriers in immunotherapy and CAR T cell therapy. Research is actively pursuing the synergistic potential of CAR T therapy alongside immune checkpoint inhibitors (ICIs), which are known to rejuvenate exhausted T cells and enhance the immunogenicity of the tumor microenvironment (93, 94). This combination is particularly promising in solid tumors, where ICIs alone have shown limited efficacy. Numerous clinical trials are evaluating the efficacy of combining CAR T cells with ICIs like PD-1 and CTLA-4 inhibitors to potentiate the anti-tumor response while aiming to mitigate immune-related adverse events (95, 96).

Moreover, the strategic use of targeted therapies such as tyrosine kinase inhibitors (TKIs) alongside CAR T cells is another area of exploration (97). TKIs can modulate key signaling pathways within both tumor cells and T cells, potentially enhancing the CAR T cells’ ability to persist and remain active in hostile tumor environments (97, 98). This approach is being tested in various hematologic and solid tumors, with early trials showing promising enhancements in CAR T cell functionality and overall survival rates (98) (NCT04257578, NCT04484012).

Additionally, other targeted therapies like DNA damage repair inhibitors, which sensitize cancer cells to immunotherapy and promote infiltration (99), and angiogenesis inhibitors, which can alter the tumor microenvironment to improve T cell infiltration and function (100), are being investigated. The combination of CAR T cells with BRAF and MEK inhibitors (101), EZH2 inhibitors (102), lenalidomide (103) and others could potentially lead to synergistic anti-tumor effects, further enhancing the efficacy of CAR T cell therapies in complex oncological landscapes.

Low-dose chemotherapy is also employed in combination with CAR T therapy as a bridging therapy, conditioning regimen, or neoadjuvant and adjuvant treatment (104). Lymphodepletion prior to CAR T cell therapy not only creates space for CAR T cells to expand but also reduces the immunosuppressive regulatory cells, thereby boosting the efficacy of CAR T cells post-infusion (105, 106). The timing, dosage, and type of chemotherapeutic agents are critical aspects currently under clinical investigation to optimize this synergy.

Radiation therapy, used concurrently with CAR T-cell therapy, is believed to promote effector T cell recruitment, remodel the tumor vasculature, enhance T cell infiltration, and alter the suppressive nature of the tumor environment (107). This combination is particularly examined in solid tumors to increase local control and potentially generate systemic immune responses (108, 109). However, fractionation, dosing, and timing of radiation should be optimized to maximize the potential therapeutic benefits while minimizing potential risks like radiation-induced T cell apoptosis.

Oncolytic viruses and bispecific T-cell engagers (BiTEs) represent innovative combination partners for CAR T cells as well. Oncolytic viruses can lyse tumor cells, alter the TME to make it more susceptible to immune cell mediated attack, and provoke an innate immune response that may prime the tumor for CAR T cell therapy (110). Multiple studies have demonstrated various degrees of success and the potential of this combinatory approach (111–113). Meanwhile, BiTEs can bridge CAR T cells to tumor cells by targeting two different antigens simultaneously, which could reduce antigen escape and enhance the specificity of the CAR T-cell response (114, 115).

In summary, combining CAR T-cell therapy with other cancer treatments such as ICIs, TKIs, and other targeted therapies is a promising frontier in oncology. While these combinations show potential in enhancing efficacy and overcoming resistance, substantial challenges remain. These include the complexity of treatment regimens, potential for increased toxicity, and the need for meticulously designed clinical trials to determine optimal dosing and scheduling. Further research and interdisciplinary collaboration are crucial to balance innovation with careful evaluation, ensuring these therapies can be safely and effectively integrated into clinical practice, ultimately improving patient outcomes.

### 2.5 Allogeneic CAR T-cell therapy: innovations for on-demand use and strategies to prevent graft-versus-host disease and rejection

Autologous CAR T-cell therapies, while highly personalized, face significant limitations, including treatment delays, complexity, and variability in T-cell quality and quantity, which can affect efficacy. These therapies involve a time-consuming process where a patient’s own T cells are harvested and engineered to express CARs, introducing accessibility issues for patients with rapidly progressing diseases or insufficient T-cell counts. In contrast, allogeneic CAR T cells, derived from healthy donors and manufactured in bulk, provide a ready-to-use solution that can be standardized, potentially leading to more consistent therapeutic outcomes across different patients. This “off-the-shelf” approach offers immediate availability, reduces manufacturing time and costs, and enables rapid deployment in acute clinical settings, making it a more accessible and cost-effective treatment option for a wider patient population **Table 4**.

**Table 4:** Comparison of allogeneic vs. autologous CAR T-Cell therapies.

Feature	Allogeneic CAR T-Cell Therapy	Autologous CAR T-Cell Therapy
**Source of T Cells**	T cells are derived from healthy donors.	T cells are derived from the patient themselves.
**Manufacturing Time**	Shorter preparation time, as cells are pre-manufactured.	Longer preparation time, cells must be collected and engineered per patient.
**Cost**	Potentially lower cost due to the "off-the-shelf" nature.	Higher cost due to the personalized manufacturing process.
**Risk of Rejection**	Higher, due to potential immune reaction against donor cells.	Lower, as the cells are the patient’s own.
**Risk of GVHD (Graft vs. Host Disease)**	Present, requires genetic modifications to reduce risk.	Absent, as the cells originate from the patient.
**Scalability**	High, as cells can be produced in large batches.	Low, each batch is patient specific.
**Availability**	Immediate availability for use in acute settings.	Requires weeks to months for cell preparation.
**Clinical Applications**	May be limited by immune compatibility issues.	Broad applicability, especially in approved indications.

However, the application of allogeneic CAR T cells introduces the risk of graft-versus-host disease (GVHD), a serious complication stemming from the donor immune cells attacking the recipient’s body. To mitigate this risk, sophisticated genetic engineering strategies are being employed. These include the knockout of the T-cell receptor (TCR) alpha chain gene or beta chain gene (116–118) to prevent the recognition of host cells by the infused CAR T cells, the use of virus-specific T cells with more restricted TCR repertoire as a source of generating allogeneic CAR T cells (119), and the use of non-αβ T cells, such as NK cells, invariant NK (iNKT), γδ T cells, or CD4/CD8 double negative T cells to engineer CAR cells (120–124).

Allogeneic CAR T-cell therapies face significant challenges in ensuring their evasion of the host immune surveillance and effective expansion and persistence in patients–pivotal for achieving sustained antitumor responses. To specifically target this issue, researchers have innovated by integrating an alloimmune defense receptor (ADR) which targets activated T and NK cells expressing the 4-1BB activation marker, thereby evading immune-mediated rejection while maintaining antitumor efficacy (125). This approach not only enhances CAR T-cell persistence but also minimizes the risk of graft-versus-host disease. Additionally, optimizing lymphodepletion and refining gene editing for T-cell robustness are crucial for overcoming these expansion and persistence challenges in allogeneic CAR T-cell therapy (117, 126).

The transformative potential of allogeneic CAR T-cell therapy lies in its ability to provide standardized, ready-to-use treatments that can be rapidly deployed. Nonetheless, this approach necessitates advanced genetic engineering to prevent immune rejection and GVHD. Addressing these challenges involves navigating complex ethical and regulatory concerns while ensuring long-term safety and efficacy through rigorous clinical trials. Balancing innovation with patient safety will be essential to realize the full potential of allogeneic CAR T cells, making them a viable option for a broader spectrum of patients globally.

### 2.6 Advanced strategies to mitigate toxicities in CAR T-cell therapy: engineering safety switches and enhancing control

In the quest to overcome the toxicities associated with CAR T-cell immunotherapy, several innovative strategies are being employed to enhance safety and control. The integration of “safety switches”, such as inducible caspase-9 (127–130) and antibody-dependent cell-mediated cytotoxicity (ADCC) switches (131), enables the rapid elimination of CAR T cells in the event of severe side effects. Additionally, small molecule-based safety switches have been developed, allowing clinicians to rapidly deactivate the cells if adverse effects occur (132–134). Another promising approach is the use of Split, Universal, and Programmable (SUPRA) CARs, which divide the CAR system into two distinct components that must interact for activation. This design enhances control, enables more precise targeting, and reduces unintended T-cell activation (135, 136).

Similarly, Dual CARs employ a strategy where CAR T cells are engineered to express two distinct receptors, requiring recognition of two specific antigens for activation. This dual recognition system enhances the specificity of CAR T cells, significantly reducing the risk of off-target effects and increasing the safety profile of these therapies (137). Control of CAR expression is achieved using inducible promoters (138, 139) or drug-responsive elements (140, 141), and innovative methods like sound (142) or light (143) activation, allowing for precise temporal and spatial control over CAR expression. This controlled approach helps to minimize the risk of overactivation and associated toxicities.

Local delivery of CAR T cells, which involves administering these cells directly to the tumor site, minimizes systemic exposure and reduces the risk of widespread toxicities often associated with broader systemic administration (89, 144). Prophylactic use of medications such as tocilizumab and anakinra is explored to preemptively reduce the severity of cytokine release syndrome (CRS) and neurotoxicity (145–147). Techniques like biomarker monitoring (148) and fractionated dosing (149) are under investigation to better predict and manage toxic responses by moderating CAR T-cell activity. Furthermore, advancements in cellular engineering, such as CRISPR/Cas9 gene editing, are aimed at enabling CAR T cells to resist activation by certain cytokines that contribute to toxicities (150, 151). Mouse models are also being developed to study the pathogenesis of CRS and neurotoxicity in CAR T-cell therapy, aiding in the identification of key inflammatory pathways and the testing of new safety interventions (14, 152, 153).

The implementation of advanced safety strategies such as safety switches, controlled activation systems, and localized delivery methods represents a significant advancement in mitigating the inherent toxicities of CAR T-cell therapy. These innovations offer enhanced control over CAR T-cell function, potentially reducing severe side effects and improving patient safety. However, these sophisticated mechanisms also introduce greater complexity into therapy design and application, which could impact both the reliability and cost of treatments. The integration of such advanced features necessitates rigorous clinical trials to confirm their efficacy and safety, alongside ethical considerations regarding access and cost. As research progresses, the challenge will be to refine these technologies to ensure they enhance therapeutic outcomes without compromising efficacy, paving the way for safer, more effective CAR T-cell therapies accessible to a wider range of patients.

### 2.7 Optimizing production, enhancing accessibility and reducing cost: innovative strategies in CAR T-cell therapy manufacturing

To tackle the challenges of manufacturing and accessibility in CAR T-cell therapy, researchers are deploying multiple innovative strategies aimed at streamlining production, enhancing efficiency, and reducing costs. Automated manufacturing systems are a pivotal advancement, utilizing closed-system bioreactors that standardize the production process, diminish the risk of contamination, and minimize labor costs. These systems can significantly cut down on the time required to produce therapeutic doses of CAR T cells (154–156), **Table 5**. Point-of-care manufacturing involves the development of compact, on-site production units within hospital settings, which reduces logistical complexities associated with the transport of cellular materials and shortens the turnaround time from collection to infusion. This approach not only speeds up the treatment process but also aims to lower overall therapy costs (157, 158).

**Table 5:** Comparison of automated vs. manual CAR T-Cell manufacturing techniques.

Feature	Automated Closed Systems	Manual Processing Methods
**Scalability**	High scalability due to standardized processes. Can handle larger batches and multiple productions simultaneously.	Limited scalability. Labor-intensive and harder to scale up due to reliance on skilled technicians.
**Reproducibility**	High reproducibility with less variability between batches due to controlled, consistent processes.	Lower reproducibility with potential for greater variability due to human involvement in processing steps.
**Cost-effectiveness**	Potentially more cost-effective in the long run due to reduced labor costs and increased throughput. High initial investment in equipment and setup.	Less cost-effective for large-scale production due to higher labor costs and longer processing times. Lower initial investment.
**Quality of CAR T Cells**	Consistent quality with automated monitoring and standardized protocols. Minimizes human error and maintains strict environmental and process controls.	Quality can vary; highly dependent on the skill and consistency of the personnel involved. More susceptible to human error.

Advanced cell expansion techniques are being developed to improve the yield and functionality of CAR T cells, including optimizing the growth media (159, 160) and conditions in bioreactors (161). Artificial Intelligence (AI) and Machine Learning (ML) have transformative potential in optimizing the production, enhancing accessibility, and reducing the costs of CAR T cell therapy. AI can streamline manufacturing processes through automation and precise control, ensuring consistency and quality while reducing labor costs (162–164). Machine learning models can predict patient outcomes from pre-infusion transcriptomes, outperforming traditional methods (165). Additionally, neural networks help design CAR constructs with optimal signaling motifs, streamlining the development and enhancing the accessibility of these therapies (166).

Economic analysis is integral to the strategies for optimizing CAR T-cell therapy manufacturing, enhancing accessibility, and reducing costs (16, 167). The high cost of CAR T-cell therapies primarily stems from the complexity of the production processes and the personalized nature of the treatments. Strategies to reduce these costs include streamlining manufacturing protocols, employing automated systems, and developing scalable batch processes which can reduce labor costs and minimize errors (16). Economic benefits also arise from shortening production times and reducing the footprint of manufacturing facilities through point-of-care production technologies (157, 158). Furthermore, adopting non-viral gene transfer methods and utilizing less costly reagents can significantly cut production expenses (48). By lowering the cost of goods and improving manufacturing efficiency, these therapies can become more accessible, particularly in low- and middle-income countries, where the burden of treatment costs is most pronounced. Engaging in cooperative strategies with global partners and governments to establish local manufacturing facilities can also reduce transportation costs and tariffs, further driving down prices and expanding access (168, 169). These economic considerations are essential for the widespread adoption of CAR T-cell therapies and require ongoing innovation and investment to ensure that these life-saving treatments are affordable and available to all patients in need, regardless of geographic location.

Efforts to streamline regulatory approvals for new manufacturing facilities and methods are crucial. Engaging with regulatory bodies to simplify and expedite the review and approval processes can significantly decrease the time and financial burden associated with bringing CAR T-cell therapies to market (170–172). Additionally, addressing regulatory and ethical considerations is essential, especially as CAR T-cell therapies involve complex genetic manipulations and personalized treatment protocols. Regulatory frameworks must ensure patient safety, manage ethical concerns related to genetic editing, and handle the implications of using donor cells in allogeneic therapies. Ethical considerations also extend to ensuring equitable access to these potentially life-saving therapies, preventing disparities in healthcare outcomes. Collaborative dialogues with ethicists, patient advocacy groups, and regulators are necessary to navigate these aspects effectively, ensuring that CAR T-cell therapies are not only scientifically sound but also socially responsible and accessible to all segments of the population.

In summary, the innovative strategies aimed at optimizing the production, enhancing accessibility, and reducing the cost of CAR T-cell therapies present significant advancements in the field. Automated manufacturing systems, point-of-care production units, and global manufacturing networks have the potential to standardize treatments, reduce production time, and make therapies more accessible, especially in underserved regions. However, these advances bring complexities, including high initial costs, operational challenges, and significant regulatory hurdles. Moreover, the integration of AI and machine learning promises further optimization but requires careful implementation to ensure quality and efficacy are maintained. As the field progresses, a balanced approach that addresses these technological, regulatory, and ethical challenges will be crucial for realizing the full potential of CAR T-cell therapies, making them a viable option for a broader range of patients globally. This comprehensive strategy will need to continue evolving, guided by ongoing research and adaptation to new insights and technological advancements.

## 3 Discussion

This review underscores the transformative strides being made in CAR T-cell therapy, with a particular emphasis on overcoming limitations that have restricted its application beyond hematologic malignancies. As we venture into novel territories, such as solid tumors and non-cancerous diseases, the synthesis of interdisciplinary advances has paved the way for potential breakthroughs, yet it also presents a complex landscape of challenges and opportunities.

### 3.1 Interdisciplinary innovation and its implications

The integration of bioinformatics, materials science, immunology, synthetic biology, genetic engineering, immunogenetics, and biomedical engineering has ushered in a new era of precision in CAR T-cell therapy. The utilization of computational tools to design CAR constructs and predict therapeutic outcomes is revolutionizing how treatments are personalized. Synthetic biology and genetic engineering are enhancing the specificity and efficacy of these constructs, while immunogenetics helps tailor therapies to individual immune system variations. Biomedical engineering contributes to the development of biocompatible materials and advanced delivery systems that improve the *in vivo* functionality of therapeutic cells. However, the translation of these complex designs from the bench to bedside necessitates innovations in manufacturing processes that can accommodate such personalized approaches at scale. Future research should focus on developing modular platforms that can be easily adapted to incorporate new discoveries and patient-specific data.

### 3.2 Economic and ethical considerations in global access

While technological advancements promise to enhance efficacy and safety, their real-world application raises significant economic and ethical questions. The high cost of these therapies remains a formidable barrier to access in low- and middle-income countries. Future initiatives should explore the development of cost-effective production methods such as the use of automated and decentralized manufacturing units. Ethically, there is a need to establish frameworks that ensure equitable access to these therapies globally, perhaps through international collaborations and policy reforms.

### 3.3 Regulatory evolution

As CAR T-cell therapies evolve, so too must the regulatory frameworks that govern their development and deployment. The rapid pace of innovation challenges current regulatory paradigms, which are often ill-equipped to handle the nuances of advanced gene and cell therapies. An ongoing dialogue between regulators, researchers, and industry stakeholders is essential to develop more adaptive regulatory approaches that can keep pace with technological advancements while ensuring patient safety.

### 3.4 Emerging areas of research

Looking forward, CAR T-cell therapies are expanding into exciting new territories. For autoimmune diseases, these therapies show promise in conditions like systemic lupus erythematous (173), multiple sclerosis (174) and type 1 diabetes (175), where they may modulate immune responses similarly to their actions against malignant cells. In the realm of aging and degenerative diseases, CAR T-cells are being investigated for their potential to modify the aging process and treat age-related ailments (176, 177). Additionally, the adaptation of CAR T-cell therapies for infectious diseases suggests a new frontier in managing chronic infections that resist conventional treatments (178). This expansion not only broadens the therapeutic potential of CAR T-cell therapies but also highlights the innovative cross-disciplinary approaches being undertaken to overcome current limitations and explore new applications.

## 4 Conclusion

The path forward for CAR T-cell therapy involves not only scientific and technological innovation but also a concerted effort to address the logistical, economic, and ethical challenges that come with such profound medical advancements. As we continue to push the boundaries of what is possible in medical science, a balanced approach that incorporates clinical needs, patient safety, and equitable access will be crucial for realizing the full potential of CAR T-cell therapies.

## Glossary

CAR T-cell Therapy: A type of cancer treatment where T cells are modified to express chimeric antigen receptors (CARs) that target specific cancer cells
Chimeric Antigen Receptor (CAR): Engineered receptors grafted onto T cells to give them the ability to target specific proteins on cancer cells
Autologous CAR T-cells: CAR T-cells derived from a patient’s own T cells
Allogeneic CAR T-cells: CAR T-cells derived from healthy donor T cells, designed for off-the-shelf use
Tumor Microenvironment (TME): The environment surrounding a tumor, including blood vessels, immune cells, fibroblasts, signaling molecules, and the extracellular matrix
Cytokine Release Syndrome (CRS): A potentially severe side effect of CAR T-cell therapy involving a large, rapid release of cytokines into the blood
Neurotoxicity: Toxicity that affects the nervous system, potentially a side effect of CAR T-cell therapy
Multiplexed CARs: CARs that target multiple antigens to reduce the likelihood of tumor escape
Modular/Universal CARs: CAR systems designed for adaptability through switchable, bispecific adaptors to target diverse antigens
SynNotch Receptors: A dual antigen recognition system where an initial antigen interaction triggers the expression of a CAR targeting a second antigen
Hybrid CARs: CARs combining elements of T-cell receptors (TCRs) and CARs to enhance targeting of cancer-specific antigens
Third Generation CARs: CARs with multiple co-stimulatory molecules, designed to enhance activation, proliferation, and antitumor efficacy
Armored CARs: Genetically modified CARs that secrete cytokines or express ligands to enhance survival and anti-tumor activity in the tumor microenvironment
CRISPR/Cas9: A gene-editing technology used to modify CAR T-cells for improved functionality and persistence
Transposon-Based Systems (e.g., Sleeping Beauty): Non-viral gene transfer methods used to integrate CAR genes into T cells
Central Memory T cells (Tcm): T cell subsets known for their longevity and robust proliferative ability, used in CAR T-cell therapies for enhanced efficacy
Stem Cell-like Memory T cells (Tscm): T cell subsets with long-term self-renewal capacity, providing durable antitumor responses
Inducible Caspase-9 (iCasp9): A safety switch that triggers CAR T-cell apoptosis upon activation by a specific drug
Antibody-Dependent Cell-Mediated Cytotoxicity (ADCC): A mechanism by which antibodies promote the destruction of target cells by immune cells
Split, Universal, and Programmable (SUPRA) CARs: CARs divided into distinct components for enhanced control and precision in targeting
Dual CARs: CAR T-cells engineered to require recognition of two distinct antigens for activation, enhancing specificity and safety
Lymphodepletion: Pre-treatment with chemotherapy to prepare the patient’s body for CAR T-cell infusion by reducing immunosuppressive regulatory cells
Oncolytic Viruses: Viruses engineered to selectively infect and kill cancer cells, used in combination with CAR T-cell therapy
Bispecific T-cell Engagers (BiTEs): Molecules that link CAR T-cells to tumor cells by targeting two different antigens, enhancing CAR T-cell specificity and activity
Graft-versus-Host Disease (GVHD): A condition where donor immune cells attack the recipient’s body, a risk in allogeneic CAR T-cell therapy
Alloimmune Defense Receptor (ADR): A receptor integrated into CAR T-cells to target and eliminate activated host immune cells, preventing rejection
Checkpoint Inhibitors (ICIs): Drugs that block proteins which inhibit T cell activity, used in combination with CAR T-cell therapy to enhance antitumor response
Tyrosine Kinase Inhibitors (TKIs): Drugs that inhibit enzymes involved in signaling pathways, enhancing CAR T-cell persistence and activity
Bioinformatics: The application of computational tools to analyze biological data, crucial for designing CAR constructs and predicting outcomes
Artificial Intelligence (AI): Technology used to optimize manufacturing processes and predict patient outcomes in CAR T-cell therapy
Economic Analysis: Assessing the cost-effectiveness of CAR T-cell therapies to enhance accessibility and affordability
Regulatory Frameworks: Guidelines and standards set by regulatory bodies to ensure the safety and efficacy of CAR T-cell therapies
Point-of-Care Manufacturing: On-site production of CAR T-cells in hospital settings, reducing logistical complexities and treatment delays
Nano-Carriers: Nanotechnology-based delivery systems to enhance CAR T-cell migration, infiltration, and functionality in tumors
Senolytic CAR T-cells: CAR T-cells designed to target and eliminate senescent cells, potentially used in treating age-related diseases
